# Two year post-menarche rule for bracing: myth or reality?

**DOI:** 10.1186/1748-7161-8-S2-O14

**Published:** 2013-09-18

**Authors:** Stefano Negrini, Sabrina Donzelli, Monia Lusini, Salvatore Minnella, Fabio Zaina

**Affiliations:** 1 University of Brescia - IRCCS Don Gnocchi, Milan, Italy

## Background

The SRS criteria for bracing studies proposes the limit of two years post-menarche (2YPM). This is also frequently used for bracing in the clinical everyday world.

## Purpose

To verify whether the 2YPM rule for bracing is appropriate in clinics, we aimed to answer the following four questions:

Is bone growth (according to Risser) almost completed?

Is stature growth ended?

Has scoliosis definitively finished progressing?

Are bracing results significantly reduced?

## Methods

We consecutively developed four studies from our clinical prospective database, including 5,142 Females with Idiopathic Scoliosis Patients below the age of 20 years.

**Table 1:** 

Study	Design	Inclusion criteria	Population	Outcome
1	Cross-sectional	All FISP with an x-ray at 2YPM	1102 patients Age 13.0±1.8 29.4±12.4° Cobb	European Risser staging

2	Prospective	All FISP followed-up from Menarche to end of growth (Risser 5 or twice Risser 4 in 12 months)	263 patients Age 13.1±1.1 35.2±11.1° Cobb	Height growth

3	Prospective	All FISP with at least two x-rays after 2YPM	902 patients with 2110 x-rays Age 15.0±1.1 31.8±12.4° Cobb	Progression

4	Prospective	All FISP braced after Menarche followed-up to the end of growth	230 patients Age 13.6±1.7 34.1±10.3° Cobb	Results of bracing

Classical parametric statistics have been used.

## Results

At 2YPM:

Risser stage is below 3 in 15.7%, and is still 0 in 2.4%.

Growth is not ended and is still decreasing (2.5 cm/y): 5% of patients have 4 cm of residual growth, 12% 3 cm and 25% 2 cm. End of growth is reached around 48 months post-menarche.

Scoliosis has not yet finished progressing, particularly in the third year, but also in the subsequent six months.

There is no change in the positive results of bracing before and after 2YPM, either for clinical-radiographic results and for percentage of patients improved/worsened.

## Conclusions and discussion

These results suggest the need to carefully check the usefulness of the 2YPM rule for clinical studies. While reaching clinical therapeutic decisions in front of single patients, 2YPM is not a reliable parameter, at least not in Italy.
